# Microbial Eukaryotes in Oil Sands Environments: Heterotrophs in the Spotlight

**DOI:** 10.3390/microorganisms7060178

**Published:** 2019-06-19

**Authors:** Elisabeth Richardson, Joel B. Dacks

**Affiliations:** 1 Department of Biological Sciences, University of Alberta, Edmonton, AB T6G 2E9, Canada; 2 Division of Infectious Disease, Department of Medicine, University of Alberta, Edmonton, AB T6G 2G3, Canada

**Keywords:** Oilsands, microbial eukaryote, 18s, microbial ecology, HNF, reclamation, molecular ecology, remediation, meta-genomics

## Abstract

Hydrocarbon extraction and exploitation is a global, trillion-dollar industry. However, for decades it has also been known that fossil fuel usage is environmentally detrimental; the burning of hydrocarbons results in climate change, and environmental damage during extraction and transport can also occur. Substantial global efforts into mitigating this environmental disruption are underway. The global petroleum industry is moving more and more into exploiting unconventional oil reserves, such as oil sands and shale oil. The Albertan oil sands are one example of unconventional oil reserves; this mixture of sand and heavy bitumen lying under the boreal forest of Northern Alberta represent one of the world’s largest hydrocarbon reserves, but extraction also requires the disturbance of a delicate northern ecosystem. Considerable effort is being made by various stakeholders to mitigate environmental impact and reclaim anthropogenically disturbed environments associated with oil sand extraction. In this review, we discuss the eukaryotic microbial communities associated with the boreal ecosystem and how this is affected by hydrocarbon extraction, with a particular emphasis on the reclamation of tailings ponds, where oil sands extraction waste is stored. Microbial eukaryotes, or protists, are an essential part of every global ecosystem, but our understanding of how they affect reclamation is limited due to our fledgling understanding of these organisms in anthropogenically hydrocarbon-associated environments and the difficulties of studying them. We advocate for an environmental DNA sequencing-based approach to determine the microbial communities of oil sands associated environments, and the importance of studying the heterotrophic components of these environments to gain a full understanding of how these environments operate and thus how they can be integrated with the natural watersheds of the region.

## 1. Introduction

**Figure 1:**
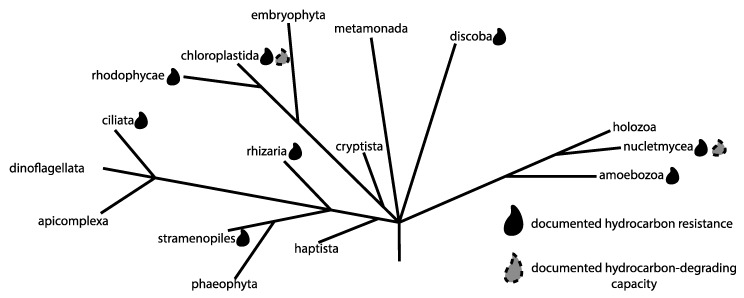
Diversity of eukaryotes, based upon Adl et al. [1]. Groups with noted resistance to hydrocarbons, or ability to degrade hydrocarbons, are indicated. It is important to note that not all taxa within the indicated groups exhibit these traits, as the tree covers all eukaryotic diversity and is therefore at extremely low taxonomic resolution. The well-known multicellular eukaryotes, i.e., animals, plants, and fungi are found within the Holozoa, Chloroplastida, and Nucletmycea respectively. These same groups also contain large numbers of microbial taxa.

The term “Eukaryote” is often considered synonymous with multicellular organisms such as animals, plants, and fungi. However, multicellular diversity only captures a minor fraction of overall eukaryotic diversity (Figure 1). There is tremendous insight to be gained from studying the diversity of microbial eukaryotes in nature, both from an ecological and a cell biological perspective. In this review, we consider the impact of high-throughput sequencing on our understanding of microbial eukaryotic species and communities in the context of hydrocarbon-associated environments, and in particular the oil sands of Northern Alberta.

## 2. Protist Diversity and Environmental Abundance: New Technology Uncover New Realities

With the advent of environmental DNA (eDNA) and high-throughput sequencing analyses of microbial community composition, the opportunity to understand the diversity and abundance of eukaryotes has exploded [1]. Though the development of this new technique has brought technological challenges, reviewed in Thomsen inter alia [2], important information has been obtained from all manner of global environments that has forced microbiologists to re-examine some widely held preconceptions about microbial eukaryotic diversity and abundance. In this review, we consider all microbial eukaryotes, also known as protists, including those from groups which also include multicellular organisms such as Opisthokonta and Chloroplastida [1]. 

Importantly, studies of eukaryote-specific community composition face challenges which do not impact studies of the prokaryotic microbial communities of the same environments [3]. Eukaryotes have multiple protective membranes and a nucleus before the genome can be accessed, and may potentially possess shells, scales or other cell protections that hinder cell lysis [1]. Bacteria are smaller and do not have a nucleus, making their genomes more easily accessible, and metagenomic samples contain only a small percentage of eukaryotic DNA [4]. Because of the additional difficulties associated with carrying out high-throughput environmental DNA studies on eukaryotes, information on eukaryotic microbial communities has lagged behind that of bacteria. However, due to increased sequencing effort and extensive curation of eukaryote-specific gene databases, it is feasible to analyze bacterial and eukaryotic communities within a sample simultaneously and retrieve broadly comparable results [5,6]. Amplicon-driven studies, using taxonomically informative genes obtained via eukaryotic-specific primers most commonly the 18S ribosomal subunit [7], are now effectively mapping protist diversity, abundance, and microbial ecology on a global scale. Researchers in this field are also in the process of developing unified protocols for protist taxonomic classification via eDNA, most recently the EukRef initiative [8,9]. This vast and expanding field is far beyond the scope of this review, but see Massana et al. [10] for a review of protistan diversity in eDNA experiments. Nonetheless, we include here a few vignettes illustrating the types of insights possible through this approach. 

Firstly, it is not accurate to assume that the environmental factors driving bacteria and protists are the same. A 2013 analysis of protists in soil by Bates et al. [7] sampled a global selection of soil and biome types. One of the most notable discoveries was that, unlike bacteria, by far the most important explanatory variable predicting community composition for protists was soil moisture content. Soils from the Dry Valleys in the Antarctic had similar protist communities to the Mojave Desert while the humid Peruvian jungle clustered alongside the Caribbean island of Puerto Rico.

It is not accurate to assume the abundance of taxa or their function in the microbial community based on the known properties of the environment. The same paper also identified a high percentage of Apicomplexa in the soil, a result mirrored in a study of soil transcriptomes by Geisen et al. [11]. This latter work, which focused on determining the metabolically active portion of the protist community, uncovered a massive and overlooked community of parasites which appeared to be functional and thriving, and not dormant in cyst form as was previously assumed. 

Further studies have found that organisms that have long been known to science, but previously thought to be rare and relatively unimportant, can actually be highly abundant and had simply been undetected. The Tara Oceans project showed that diplonemids, a group of organisms within the Excavata, far from being rare, actually account for a large proportion of marine protist biomass. They likely have a vital role in nutrient cycling in the open oceans [12]. 

As initiatives such as the EukREF [8] and the earth microbiome project [13] come online, with the goal of comprehensively mapping microbial diversity in all environments, there are bound to be exciting surprises ahead.

## 3. Protists and Anthropogenically Impacted Environments

One of the goals of global biodiversity assessment is to set a baseline to assess change in microbial populations over time. While climate change is a key area of inquiry, human activity also impacts environments directly. Of particular interest, for economic and environmental reasons, is the petroleum industry. 

Extraction, processing, and transport of petroleum products together are a global industry worth trillions of dollars, and despite global efforts to wean societies from dependence on fossil fuels, they still comprise an enormous part of the global economy and trade [14]. Extraction of hydrocarbons from underground is traditionally understood as drilling and siphoning of liquid oil deposits—however, as conventional oil fields run low, efforts are increasingly focused on non-conventional sources such as shale oil, fracking, and oil sands [14]. Much of the information we have around how eukaryotic communities respond to oil exposure involve accidental releases of hydrocarbons in transit; for example, oil spills from tankers or pipelines into the ocean. The largest oil spill study followed the 2010 accidental release of billions of barrels of oil into the Gulf of Mexico from the Deepwater Horizon well off the coast of Louisiana, a research effort that is still ongoing [15]. The Gulf of Mexico Research Initiative provided substantial experimental data on algal responses to oil exposure. The results varied; some show differing responses down to the species level with no overall trend toward prokaryotes or eukaryotes as more sensitive or resistant [16], whereas other studies show that within eukaryotes, the size of cells and evolutionary derivation may affect hydrocarbon resistance [17]. 

Most studies of how protists are involved in chronic hydrocarbon contamination utilize ‘before and after’ studies comparing protist communities before and after disturbances occur. For example, protist community changes have been evaluated in this manner for soils associated with chronic hydrocarbon pollution in Brazil [18] and marine environments surrounding oil rigs in Norway [19]. In both of these cases, substantial changes in community structure were found, with lower community diversity in the disturbed compared to undisturbed environments. In the case of the Brazilian soils, only ciliates were evaluated, and the group *Colpodea* spp. was identified as being particularly pollution-resistant [18]. In the case of the Norwegian oil rigs, several indicator taxa for disturbance were identified, including multiple ciliate species [19]. As eDNA studies become more prevalent, particularly in the context of evaluating protist responses to hydrocarbons, the list of known hydrocarbon-resistant, hydrocarbon-susceptible, and hydrocarbon-degrading heterotrophic nanoflagellates is certain to grow.

Though the before and after studies provide valuable information into how protists, particularly algae, are affected by exposure to oil and additional chemicals such as the dispersant Corexit [16,20,21,22], the environmental pressures associated with long-term hydrocarbon exposure are very different and may well lead to lasting effects on community composition and cellular adaptations. To understand the effect of hydrocarbons in the long-term, some exciting study environments are the natural bitumen-associated environments and reclamation sites of Northern Alberta, including the boreal forest region, tailings ponds containing the waste from oil sands extraction, and reclamation sites. 

## 4. The Albertan Oil Sands

The Northern Alberta Oil Sands deposits consist of rocky sand suspended in a thin layer of bitumen and cover an enormous stretch of the boreal forest in Northern Alberta and Saskatchewan [23]. The boreal forest region of Canada is a circumpolar forested area that ranges from the Yukon through to Ontario and Quebec [23]. It is known for not only its vast forested area but the abundance of lakes, streams, and rivers [24] and is notable for its extreme temperature variations, which can range from +30 °C in the summer to −40–50 °C in the winter. 

The oil-bearing sands range from tens of meters beneath the surface to rocky outcrops leaking into the local watershed, and also by the massive oil sands mining processes that dominate discussion of the boreal forest landscape. In the extraction process, the bitumen layer is washed from the sand, either in place underground at steam-assisted ground drainage sites or in an industrial site in the case of oil sands mining, and this extracted bitumen is refined into usable crude oil [23]. Oil sands mining results in the production of solid and liquid waste, but the liquid waste, known as tailings, is particularly environmentally challenging. Reclamation efforts aimed at obtaining healthy aquatic environments that can be integrated into the local watershed from tailings and tailings ponds are currently underway, and extensively monitored and researched by industry and governmental regulators [25]. 

## 5. Protists in the Oil Sands Region

The oil sands region is particularly interesting for studying the microbial ecology of an anthropologically influenced environment. Bitumen is a naturally occurring compound in the local environment, and species have presumably adapted to its presence as detected in freshwater and soils [26]. One would expect a certain amount of resilience from the microbial communities surrounding oil deposits, but the extent to which growth in the presence of hydrocarbons might confer a physiological and community resistance to survival in mine tailings remains unknown. Ecological studies of the interactions between bitumen and microbial communities in northern Alberta are limited, and most have been performed in the context of establishing a baseline for reclamation of mining-influenced environments. Yergeau et al. [27] used 454 sequencing to survey the bacterial communities found in natural streams in the Albertan region compared to those in bitumen-containing soils in the riverbed. They found significant differences between bacterial communities of bitumen-associated and non-bitumen-associated streams. The authors suggested a considerable difference in the community structure that likely persisted through multiple trophic levels. Reid et al. [28] studied metatranscriptomics of hydrocarbon-rich freshwater sediments in the Athabasca oil sands region to determine a baseline of microbial processes before the influence of oil sands mining. Though they did not distinguish between protists and bacteria, they focused on biogeochemical processes most commonly associated with bacterial communities such as nitrogen fixation—and, as noted previously, metatranscriptomic analyses tend to be dominated by bacterial sequences. They found genes associated with the metabolism of nitrogen, sulfur, and methane, as well as hydrocarbon degradation, suggesting that the presence of hydrocarbons was integrated into the sediment ecosystem and the community structure was adapted to its presence even outside of the influence of oil sands mining. Similarly, Wong et al. [29] surveyed the bacterial communities of bitumen outcrops in Northern Albertan streams using 16S/18S rRNA sequencing. These outcrops, where heavy bitumen is exposed to the stream directly, reach temperatures of up to 55–60 °C when exposed to sunlight. The Wong et al. [29] study identified a community of hydrocarbon-degrading microbes that included a substantial thermophilic fungal component. This shows that microbial eukaryotes are an integral part of the hydrocarbon ecosystem in Northern Alberta; fungi, in particular, are excellent candidates for bioremediation as they contain the majority of described thermophilic (capable of surviving in 50+ °C temperatures) taxa and also have been identified as capable of degrading hydrocarbons in vitro and in situ [30,31]. 

Studies of the core bitumen deposits themselves have not yielded much information on microbial eukaryotes; to date, there has been no published successful recovery of 18S amplicons from within these environments [32]. However, the bacterial communities within the bitumen have been analyzed from cores taken from bitumen deposits using 16S rRNA amplicon sequencing and metagenomics [33]. The most surprising result from these studies has been a considerable contribution to nutrient cycling from an abundant aerobic bacterial population; aerobic taxa such as *Pseudomonas* and *Acinetobacter* were dominant in 16S samples and genes associated with oxygen-dependent metabolic processes were common in the metagenomes [34,35]. Core bitumen deposits might initially appear to be too challenging for eukaryotic communities; however, amplicon studies of microbial eukaryotes have found thriving communities in extremely challenging environments, including deep in Lake Baikal, in the Dry Valleys of Antarctica, and even in water droplets precipitated into clouds [36,37,38]. Since initial assessments of the environmental conditions of bitumen have been revised, it would be well worth revisiting the issue of potential eukaryotic communities. 

## 6. Tailings Ponds

**Figure 2:**
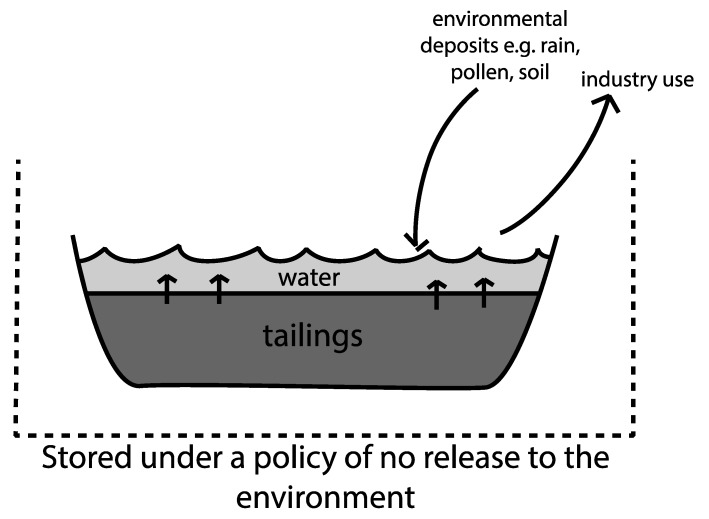
Simplified schematic of material flow between the local environment and a filled tailings pond.

Bitumen deposits are naturally mixed with sand and soil in the ground, and for the bitumen to be prepared for processing, it is separated from the soil using detergent and water at a high temperature [23]. The liquid waste, i.e., tailings, that is used in this process is then diverted for storage, as it cannot be returned to the environment due to its exposure to industrial chemicals [39]. This mixture of water from the extraction process, residual bitumen, silt, surfactants, naphthenic acids, heavy metals, and salts, is currently stored in structures known as tailings ponds. They generally consist of the semi-settled, partially solid tailings and an overwater cap produced from the water displaced from the settling tailings. This overwater is periodically siphoned off and reused for further bitumen extraction [32]. Fresh tailings from the extraction process (fluid fine tailings, or FFT), gradually settles over time into thicker, mature tailings (MFT) and a water cap [39]. Tailings ponds can be 40–50 m deep and cover multiple square kilometers; there are an estimated billion m^3^ of tailings currently stored in Northern Alberta mining sites [32]. These tailings ponds covered 77 km^2^ of the landscape as of 2013, and current oil sands mining operations produce an additional 1 million m^3^/day of fresh tailings [40,41]. Since this waste cannot be released into the local watershed until it is deemed to be reclaimed―and therefore no longer potentially harmful to the local flora and fauna―the footprint of these tailings ponds is currently increasing [32]. The components of fluid fine tailings (FFT) that are of greatest concern, and most extensively researched, are residual hydrocarbons, naphthenic acids, heavy metals, and salts [42]. Figure 2 gives a simplified diagram of the water inputs and outputs into a capped tailings pond.

The ecology of tailings ponds is an important question for reclamation. The initial studies of tailings pond ecology through high-throughput sequencing focused on bacteria, as bacterial methanogenesis is responsible for the release of enormous quantities of greenhouse gases—at its peak, the tailings pond Mildred Lake Settling Basin was releasing 40 million liters a day of methane into the atmosphere due to its bacterial community [42]. These studies, summarized in Foght et al. [32] and Siddique et al. [42] inter alia, show that there was a diverse bacterial community and extensive nutrient cycling involving anaerobic and aerobic processes.

The first indication that eukaryotes may be present in tailings ponds was reported in Saidi-Mehrabad et al. [43], in a paper that focused on methanogenic and methanotrophic bacteria present in enrichment cultures created from tailings pond samples. They noted the presence of an amoeba growing to up to 40% of DNA abundance in metagenomes of these enrichment cultures after 48 h, apparently feeding on the bacteria. However, though they identified the amoeba as most closely related to the known species *Protacanthamoeba bohemica*, they did not determine its origin or definitively show it came from the tailings pond sample rather than post-collection contamination [43]. *P. bohemica* was originally identified as a parasite of freshwater fish and has since been isolated in drinking water and in environmental screens of freshwater [44,45]; switching between parasitic and free-living heterotrophy has been noted in other amoebae such as the human pathogen *Naegleria fowleri* [46]. This suggests that *P. bohemica* would theoretically be capable of surviving in a low-light and potentially nutrient-poor environment like tailings as well as freshwater, but it is not conclusive. Amoebae have also been noted for their ability to degrade hydrocarbons in mesocosm experiments [47]. 

**Figure 3:**
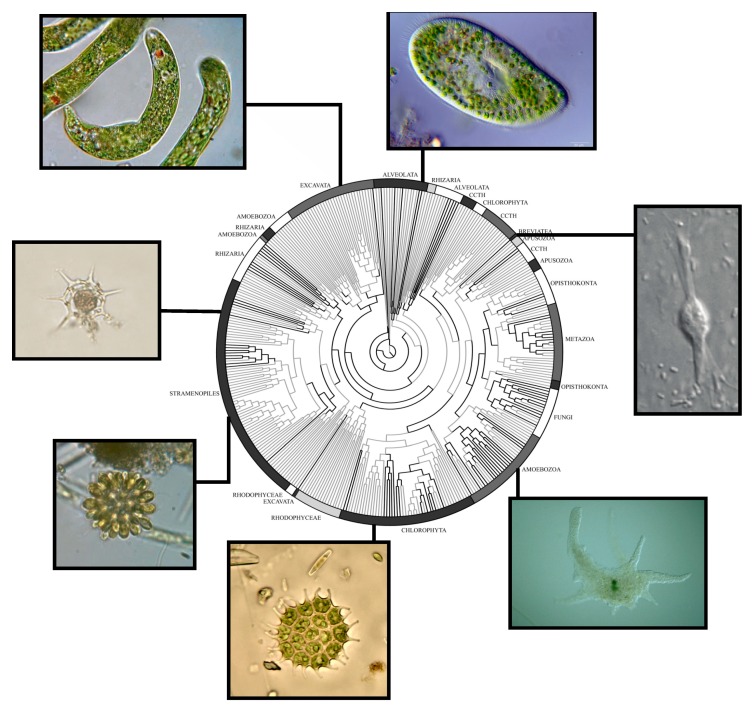
Diversity of eukaryotes identified in oil sands tailings ponds. Tree is derived from pplacer, with a universal backbone of eukaryotes based on the 18S rRNA ribosomal gene (modified with permission from Aguilar et al. [48]). Microscopy image of Breviatea derived (with permission) from Walker et al. [49]. Other Microscope images are reproduced under Creative Commons licenses from the following authors (clockwise from top left): Deuterostome; Anatoly Mikhaltsov; Tsukii Yuji; U.S. Environmental Protection Agency; Deuterostome; Minami Himemiya.

The most comprehensive study of microbial eukaryotes in tailings ponds comes from Aguilar et al. [48]. In this study, the eukaryotic community of the water cap of Mildred Lake Settling Basin (MLSB) and soft, anoxic tailings sediments of West-in Pit (WIP), two tailings storage facilities, were sampled. This study used 18S-optimised amplicon sequencing to ensure the maximum possible signal from the eukaryotic cells found within these tailings, but sequences identifiable as eukaryotes were sparse—to ensure the most accurate possible classification of this small eukaryotic sample, the authors used phylogenetics to determine how closely the extracted sequences were related to known species (Figure 3). The vast majority of the extracted sequences were heterotrophic, from the groups Rhizaria, Ciliata, and Fungi, consistent with the low-light and low-oxygen environment [48]. However, there was a notable presence of sequences from majority photosynthetic clades such as Euglena and Chlorophyta [48]. While the number of sequences extracted was too small for identification of any trophic webs or ecological trends associated with eukaryotes, the consistent detection of a eukaryotic signature in the three samples of tailings sediments argues against the eukaryotic signal merely being artifact and instead strongly shows a eukaryotic community in this environment. 

The authors also mined metagenomes produced from the MLSB sediments and also the overwater, taken from the thin oxic layer at the top of the water cap, for eukaryotic sequences. Metagenomic studies tend to be dominated by bacterial DNA and combined with the low relative abundance of eukaryotes in this environment even fewer eukaryotic sequences were identified. Those that were present included heterotrophs, phototrophs, and also some sequences classified as Metazoa, suggesting the presence of zooplankton or insects [48]. However, these sequences may not have been derived from living organisms and therefore cannot be used to make any ecological conclusions. However, it is notable that a substantial percentage of the initial protist diversity identified in studies of hydrocarbon-associated water bodies are heterotrophic; heterotrophic nanoflagellates (known as HNFs in most limnological studies) have traditionally been understudied compared to their photosynthetic algal counterparts [50,51]. 

## 7. In Vitro and In Situ Assessments of Protists in Reclamation Environments

Experiments into reclamation and remediation of oil sands waste have been going on almost as long as mining in the area. Though land previously associated with oil sands mining has in some cases been certified as reclaimed, it is a testament to the difficulty of reclaiming FFT that there is no scientifically or legally recognized strategy of re-integrating the liquid in tailings ponds back into the local watershed [40]. Some of the earliest studies of hydrocarbon reclamation focused on the in vitro effects of hydrocarbons on protists, while in situ mesocosm studies allowed small-scale reclamation methods to be tested in field environments. Both of these methods have contributed considerably to our understanding of the effects of hydrocarbons and other tailings-associated contaminants over several decades; in vitro studies have shown the effects of hydrocarbons and associated contaminants on the morphology and physiology of single eukaryotic cells, while in situ studies have provided insight into how protist and algal communities are affected by the environmental challenge of these contaminants. Though there are many environmentally challenging compounds associated with oil sands reclamation, including heavy metals and salts, it is impossible to provide an exhaustive account of the literature on single-celled eukaryotes and contaminants in a single review. Accordingly, the following section focuses on in vitro studies of protists and hydrocarbons, and mesocosms associated with oil sands mining sites in Northern Alberta. 

Rogerson and Berger carried out one of the earliest studies of the effects of hydrocarbons on protists in vitro in the late 1980s [52]. They made use of electron microscopy to examine the effects of crude oil on the ciliate *Colpoda colpodium*, a common freshwater protist which has (subsequently to this study) been shown to exhibit remarkable resistance to hydrocarbons. Rogerson and Berger also noted that exposure to crude oil was not fatal to the organism, even at relatively high concentration, but did result in pronounced visible morphological changes, including a notable increase of hydrocarbon-containing inclusions and an increase of “secretory ampules” adjacent to the cell surface [52]. The inclusions could be lipid droplets, as suggested by the authors, but could also be phagosomes containing the crude oil. Regardless, the increase of both types of observed membrane-bound organelles speaks to a modification of the ciliate endomembrane-system in response to the crude oil addition. This is consistent with eukaryotes being more heavily affected by hydrocarbons than bacteria, as their cell biology is more heavily reliant on membranes and compartmentalization of cellular processes by lipid-bound structures [1]. However, other studies have suggested that, rather than being incapacitated by hydrocarbons, protists may have an active role in bioremediation. The exact process for protists’ contributions to hydrocarbon degradation is unknown; some in vitro studies have shown that inhibition of protist grazing inhibits hydrocarbon degradation [53], suggesting that protists are enhancing degradation by preying on bacteria [54], while other studies suggest that protists are degrading hydrocarbons directly [31,47]. Additionally, Gilbert et al. [55] showed that heterotrophic grazers such as ciliates may act as biological dispersants of hydrocarbons, contributing to the bioremediation of hydrocarbon contamination by dispersing the lipid droplets that can cut off oxygen to the water column and also prove lethal to macrofauna and flora. Tailings waste has also been applied in vitro to multicellular organisms, including HeLa cells and fish embryos [56,57,58]. These experiments have shown profound local toxicity to these multicellular tissues and organisms, which implies that macrofauna such as fish and plants would struggle to survive in the environmentally challenging conditions of tailings, fresh process water, and early-stage reclamation environments, though the toxicity of end-pit lake waters would certainly decrease over time due to dilution and reclamation processes. 

## 8. Protists and Oil Sands Reclamation

Similar to in vitro studies, studies into how hydrocarbons affect protists in situ have been underway since the 1980s, when oil exploration in Northern Alberta expanded as an industry [59]. These mesocosms, which have been established at various sites in Alberta, have tested how indigenous microorganisms react to being exposed to the industrial by-products of mining processes, including FFT. Most of the eukaryotes observed in these studies are phototrophs; primary production is essential for nutrient cycling [60]. However, some phototrophs indigenous to oil sands process water (OSPW) may also have a role in direct bioremediation. Ruffell et al. [61] incubated 21 algal strains extracted from sites within or near oil sands mining operations with OSPW and used mass spectroscopy to determine the fraction of oil degraded by the algae in culture. In this paper, “algae” was used to mean all oxygen-producing organisms from the OSPW and therefore the experiment included eukaryotes and prokaryotes [62]. Though multiple eukaryotic algae were used in the experiment, the only species identified as a candidate for industrial bioremediation was a cyanobacterium [61]. As an aside, the different usage of the informal term “algae” is a source of potential miscommunication between two scientific disciplines relevant to the discussion of microbial eukaryotes and oil sands environments. If defined as photosynthetic, oxygen-producing microbe, the term “algae” is clearly polyphyletic. Nonetheless, it is commonly used in the limnological literature [62] by precisely this definition, and so the context of its usage is worth bearing in mind for protistologists delving into that realm. Leung et al. [59] used cell counts to observe the eukaryotic phototroph communities in multiple water bodies in the Fort McMurray region, some of which were affected by OSPW. They noted that Chlorophyta were particularly dominant in their samples, particularly in those highly affected by OSPW, and suggested that indigenous phytoplankton communities may be somewhat resistant to the presence of hydrocarbons and associated contaminants. 

In 2012, the largest reclamation site for FFT was established at the Syncrude mine site near Fort McMurray. This site, called Base Mine Lake (BML), is an example of a proposed FFT reclamation technique called wet-capping [63]. FFT from a mine site―in this case, the decommissioned tailings pond Mildred Lake Settling Basin―are covered with a layer of freshwater that is continually replenished until the tailings are settled into a thick layer and the freshwater has diluted all potential contaminants to ecologically tolerable conditions. Geophysical studies of BML have shown positive change over the last five years. Oxygen penetration into the water is comparable to natural lakes in the Northern Alberta region, and heavy metal and hydrocarbon contaminations levels have returned to levels that are well within water quality guidelines and compare to undisturbed lakes in the local area [64]. However, salinity is projected to be a risk for decades to come [64] and, due to the mixing processes common to boreal forest lakes, the water cap experiences high turbidity in the spring [65]. Studies of bacterial community structures in Base Mine Lake and other tailings pond reclamation sites in the region have shown robust bacterial communities, and there are diverse communities of bacteria present in reclamation sites contributing to nutrient cycling [66]. 

Studies of the overall community composition for bacteria and eukaryotes in Base Mine Lake are underway, and will no doubt produce extremely interesting and informative results that will aid the reclamation effort in Northern Alberta. Due to the quantity of tailings that will need to be incorporated into the environment, these end-pit lake systems are all extremely large and complex water bodies that can be considered individual ecosystems in their own rights and have a correspondingly unique biology and history. Because eDNA approaches allow us to evaluate microbial communities on a high-throughput scale, there is potential for the integration of holistic bioindicator species that can be empirically shown as influenced by the anthropogenic disturbance specific to the industrial site under reclamation. An advantage of eDNA approaches is all species―whether extremely large and distinct or tiny and relatively homogenous in appearance―are evaluated using a molecular approach and therefore ease of visual identification is no longer a relevant factor for establishing bioindicator species, leaving a gap that has the potential to be filled by heterotrophic protists, or heterotrophic nanoflagellates.

Heterotrophic protists—or, as they are often referred to in ecology, heterotrophic nanoflagellates—have been suggested, and in some cases incorporated, into industrial reclamation more frequently since the advent of widely accepted molecular methods such as eDNA for quickly evaluating community structure. Rekik et al. [67] surveyed the ciliate community of the north coast of Sfax, Tunisia, during coastal reclamation. The environment had been affected by contamination from the industrial plants in the city and was suffering from declining marine life and fish stocks. During the reclamation process, the researchers noted a high seasonal variation of ciliate populations, with distinct summer and winter assemblages. They also noted that biodiversity of ciliates increased after restoration, though biomass decreased, likely due to predation by newly restored macrofaunal populations [67]. This study demonstrates some of the benefits of including heterotrophic protists as reclamation indicators; they represent a trophic level intermediate between bacteria, which are profoundly affected by geochemistry, and macrofauna that may be too affected by local toxicity to reach measurable abundance in a reclamation context. Many heterotrophic protists are highly resilient, and can survive low light, periods of partial or complete anoxia, and the presence of toxic compounds [68]; even primarily phototrophic algae employ heterotrophic strategies in periods of nutrient limitation or low light [69]. Industrial evaluation of protist communities is now common practice in wastewater treatment plants, ever since it was established in the 1970s that “ciliated protozoa” are essential for water treatment to be successful [70]. The limitations and successes of heterotrophic protists in wastewater treatment are reviewed in Foissner et al. [70], and their most relevant conclusion in the context of oil sands reclamation is that for heterotrophic protists to be useful as bioindicators, one must understand the taxonomy and ecology of these enigmatic organisms. eDNA and other molecular approaches appear to be filling this niche, even with the undisputed decline of microscopy as a relevant technique for community ecology [3]. Phylogenetic approaches have led to the expansion of our understanding of two of the most morphologically homogenous, yet genetically diverse, of these heterotrophic clades; the fungal Microsporidia [50] and the cercozoan Glissamonada [51,71]. The explosion of identified species belonging to these clades, as well as an increased appreciation of their ecological abundance and relevance, indicates the growing potential for heterotrophic protists as underappreciated bioindicators, both of industrial disturbance and reclamation. 

Understanding the microbial ecology of multiple local undisturbed or non-hydrocarbon-affected lakes or reservoirs is also necessary to ensure that the baseline for reclamation is representative, no matter what species is used as a bioindicator. Multiple biotic and abiotic factors can affect reclamation: This may include differences in industrial processes used to produce tailings; heterogeneity of microbial communities even in the relatively restricted local environment; and unusual features of the geography found in a specific time or place. For example, in the case of Base Mine Lake, this environment was exposed to the enormous Fort McMurray forest fire in the early summer of 2016, which resulted in the burning of nearly 6000 km^2^ of boreal forest in the region [72]. Forest fires, which have long been a natural part of the prairie and boreal forest landscape, result in substantial nutrient deposition in regional lakes. This effect is most well-studied in nitrogen cycling, though there is evidence that it also causes an increase in phosphorus deposition that can, alongside the nitrogen, lead to a boost in microbial growth [73,74,75]. If one of the limiting factors for the growth of photosynthetic algae was the availability of nitrogen or phosphorus, then the 2016 Fort McMurray forest fire may result in an increased lake biomass and significantly affect microbial eukaryotic population dynamics going forward. Accordingly, it will be interesting to see whether this event is visible in the microbial dynamics in watersheds in the boreal forest region. 

## 9. Final Remarks

Reclamation of hydrocarbon-associated environments is a key issue for the coming years, as humanity’s reliance on oil is likely to persist for decades to come. Increased use of high-throughput sequencing has allowed a greater understanding of the microbial communities responsible for nutrient cycling and supporting higher trophic levels and have provided valuable insight into how these processes work both in a reclamation context and in a natural environment. It is now inescapably clear that protists are an essential component of these microbial ecosystems and should be rigorously monitored to ensure that biodiversity and ecosystem function is maintained, both in a reclamation context and in response to future environmental challenges such as a changing climate. While algae are an established and routinely measured aspect of environments in reclamation assessment, the notable presence of heterotrophic protists in diverse hydrocarbon environments suggests that closer monitoring of these organisms in future reclamation sites may be a particularly interesting line of research; if heterotrophs return early to hydrocarbon-affected environments (or, indeed, are able to survive the conditions association with industrial bitumen extraction) then they may provide exceptional insight into both cellular adaptations associated with hydrocarbon exposure and, conversely, act as bioindicators for successful or unsuccessful reclamation strategies. The resurgence of interest in heterotrophic nanoflagellates in both a reclamation context and in the natural environment is likely to prove extremely productive for our understanding of ecology as well as taxonomy. 

## References

[1] Adl S.M., Bass D., Lane C.E., Lukeš J., Schoch C.L., Smirnov A., Agatha S., Berney C., Brown M.W., Burki F. (2018). Revisions to the Classification, Nomenclature, and Diversity of Eukaryotes. J. Eukaryot. Microbiol..

[2] Thomsen P.F., Willerslev E. (2014). Environmental DNA—An emerging tool in conservation for monitoring past and present biodiversity. Biol. Conserv..

[3] Bik H.M., Porazinska D.L., Creer S., Caporaso J.G., Knight R., Thomas W.K. (2012). Sequencing our way towards understanding global eukaryotic biodiversity. Trends Ecol. Evol..

[4] Bik H.M., Sung W., de Ley P., Baldwin J.G., Sharma J., Rocha-Olivares A., Thomas W.K. (2012). Metagenetic community analysis of microbial eukaryotes illuminates biogeographic patterns in deep-sea and shallow water sediments. Mol. Ecol..

[5] Obbels D., Verleyen E., Mano M.-J., Namsaraev Z., Sweetlove M., Tytgat B., Fernandez-Carazo R., De Wever A., D’hondt S., Ertz D. (2016). Bacterial and eukaryotic biodiversity patterns in terrestrial and aquatic habitats in the Sør Rondane Mountains, Dronning Maud Land, East Antarctica. FEMS Microbiol. Ecol..

[6] Mesa V., Gallego J.L.R., González-Gil R., Lauga B., Sánchez J., Méndez-García C., Peláez A.I. (2017). Bacterial, Archaeal, and Eukaryotic Diversity across Distinct Microhabitats in an Acid Mine Drainage. Front. Microbiol..

[7] Bates S.T., Clemente J.C., Flores G.E., Walters W.A., Parfrey L.W., Knight R., Fierer N. (2013). Global biogeography of highly diverse protistan communities in soil. ISME J..

[8] del Campo J., Kolisko M., Boscaro V., Santoferrara L.F., Nenarokov S., Massana R., Guillou L., Simpson A., Berney C., de Vargas C. (2018). EukRef: Phylogenetic curation of ribosomal RNA to enhance understanding of eukaryotic diversity and distribution. PLoS Biol..

[9] Berney C., Ciuprina A., Bender S., Brodie J., Edgcomb V., Kim E., Rajan J., Parfrey L.W., Adl S., Audic S. (2017). *UniEuk*: Time to Speak a Common Language in Protistology!. J. Eukaryot. Microbiol..

[10] Massana R. (2015). Protistan Diversity in Environmental Molecular Surveys. Marine Protists.

[11] Geisen S. (2016). Thorough high-throughput sequencing analyses unravels huge diversities of soil parasitic protists. Environ. Microbiol..

[12] Flegontova O., Flegontov P., Malviya S., Audic S., Wincker P., de Vargas C., Bowler C., Lukeš J., Horák A. (2016). Extreme Diversity of Diplonemid Eukaryotes in the Ocean. Curr. Biol..

[13] Gilbert J.A., Jansson J.K., Knight R. (2014). The Earth Microbiome project: Successes and aspirations. BMC Biol..

[14] OPEC (2018). OPEC: World Oil Outlook.

[15] Colwell R.R., Leinen M., Benoit D.S., Brewer P.G., Dodge R.E., Farrington J.W., Halanych K.M., Halpern D., Hogarth W.T., Mauritzen C. The Gulf of Mexico Research Initiative: A New Research Paradigm. Proceedings of the International Oil Spill Conference Proceedings.

[16] Bretherton L., Williams A., Genzer J., Hillhouse J., Kamalanathan M., Finkel Z.V., Quigg A. (2018). Physiological response of 10 phytoplankton species exposed to macondo oil and the dispersant, Corexit. J. Phycol..

[17] Özhan K., Miles S.M., Gao H., Bargu S. (2014). Relative phytoplankton growth responses to physically and chemically dispersed South Louisiana sweet crude oil. Environ. Monit. Assess..

[18] Lara E., Berney C., Harms H., Chatzinotas A. (2007). Cultivation-independent analysis reveals a shift in ciliate 18S rRNA gene diversity in a polycyclic aromatic hydrocarbon-polluted soil. FEMS Microbiol. Ecol..

[19] Lanzén A., Lekang K., Jonassen I., Thompson E.M., Troedsson C. (2016). High-throughput metabarcoding of eukaryotic diversity for environmental monitoring of offshore oil-drilling activities. Mol. Ecol..

[20] Rahsepar S., Smit M.P.J., Murk A.J., Rijnaarts H.H.M., Langenhoff A.A.M. (2016). Chemical dispersants: Oil biodegradation friend or foe?. Mar. Pollut. Bull..

[21] Snyder R.A., Ederington-Hagy M., Hileman F., Moss J.A., Amick L., Carruth R., Head M., Marks J., Tominack S., Jeffrey W.H. (2014). Polycyclic aromatic hydrocarbon concentrations across the Florida Panhandle continental shelf and slope after the BP MC 252 well failure. Mar. Pollut. Bull..

[22] Rodriguez-R L.M., Overholt W.A., Hagan C., Huettel M., Kostka J.E., Konstantinidis K.T. (2015). Microbial community successional patterns in beach sands impacted by the Deepwater Horizon oil spill. ISME J..

[23] Canadian Association of Petroleum Producers (2018). Canada’s Oil Sands Fact Book.

[24] Brandt J.P., Flannigan M.D., Maynard D.G., Thompson I.D., Volney W.J.A. (2013). An introduction to Canada’s boreal zone: Ecosystem processes, health, sustainability, and environmental issues. Environ. Rev..

[25] Government of Alberta (2014). Responsible Energy Development Act.

[26] Mossop G.D. (1980). Geology of the Athabasca Oil Sands. Science (80-).

[27] Yergeau E., Lawrence J.R., Sanschagrin S., Waiser M.J., Korber D.R., Greer C.W. (2012). Next-generation sequencing of microbial communities in the Athabasca River and its tributaries in relation to oil sands mining activities. Appl. Environ. Microbiol..

[28] Reid T., Chaganti S.R., Droppo I.G., Weisener C.G. (2018). Novel insights into freshwater hydrocarbon-rich sediments using metatranscriptomics: Opening the black box. Water Res..

[29] Wong M.-L., An D., Caffrey S.M., Soh J., Dong X., Sensen C.W., Oldenburg T.B.P., Larter S.R., Voordouw G. (2015). Roles of Thermophiles and Fungi in Bitumen Degradation in Mostly Cold Oil Sands Outcrops. Appl. Environ. Microbiol..

[30] Aranda E. (2016). Promising approaches towards biotransformation of polycyclic aromatic hydrocarbons with Ascomycota fungi. Curr. Opin. Biotechnol..

[31] Al-Hawash A.B., Alkooranee J.T., Abbood H.A., Zhang J., Sun J., Zhang X., Ma F. (2018). Isolation and characterization of two crude oil-degrading fungi strains from Rumaila oil field, Iraq. Biotechnol. Rep..

[32] Foght J.M., Gieg L.M., Siddique T. (2017). The microbiology of oil sands tailings: Past, present, future. FEMS Microbiol. Ecol..

[33] Gieg L.M. (2018). Microbial Communities in Oil Shales, Biodegraded and Heavy Oil Reservoirs, and Bitumen Deposits. Microbial Communities Utilizing Hydrocarbons and Lipids: Members, Metagenomics and Ecophysiology.

[34] Ridley C.M., Voordouw G. (2018). Aerobic microbial taxa dominate deep subsurface cores from the Alberta oil sands. FEMS Microbiol. Ecol..

[35] An D., Caffrey S.M., Soh J., Agrawal A., Brown D., Budwill K., Dong X., Dunfield P.F., Foght J., Gieg L.M. (2013). Metagenomics of Hydrocarbon Resource Environments Indicates Aerobic Taxa and Genes to be Unexpectedly Common. Environ. Sci. Technol..

[36] Yi Z., Berney C., Hartikainen H., Mahamdallie S., Gardner M., Boenigk J., Cavalier-Smith T., Bass D. (2017). High-throughput sequencing of microbial eukaryotes in Lake Baikal reveals ecologically differentiated communities and novel evolutionary radiations. FEMS Microbiol. Ecol..

[37] Bielewicz S., Bell E., Kong W., Friedberg I., Priscu J.C., Morgan-Kiss R.M. (2011). Protist diversity in a permanently ice-covered Antarctic lake during the polar night transition. ISME J..

[38] Amato P., Joly M., Besaury L., Oudart A., Taib N., Moné A.I., Deguillaume L., Delort A.-M., Debroas D. (2017). Active microorganisms thrive among extremely diverse communities in cloud water. PLoS ONE.

[39] Charette T., Castendyk D., Hrynyshyn J., Kupper A., McKenna G., Mooder B. (2015). End Pit Lakes Guidance Document 2012.

[40] Alberta Energy Regulator (2017). Directive 085: Fluid Tailings Management for Oil Sands Mining Projects.

[41] Siddique T., Stasik S., Mohamad Shahimin M.F., Wendt-Potthoff K. (2018). Microbial Communities in Oil Sands Tailings: Their Implications in Biogeochemical Processes and Tailings Management. Microbial Communities Utilizing Hydrocarbons and Lipids: Members, Metagenomics and Ecophysiology.

[42] Mahaffey M.A., Dube D.M. (2016). Review of the composition and toxicity of oil sands process-affected water. Environ. Rev..

[43] Saidi-Mehrabad A., He Z., Tamas I., Sharp C.E., Brady A.L., Rochman F.F., Bodrossy L., Abell G.C., Penner T., Dong X. (2013). Methanotrophic bacteria in oilsands tailings ponds of northern Alberta. ISME J..

[44] Dykova I., Veverkova Fialova M., Fiala I., Dvorakova H. (2005). Protacanthamoeba bohemica sp. n., isolated from the liver of tench, Tinca tinca (Linnaeus, 1758). Acta Protozool..

[45] Buse H.Y., Lu J., Struewing I.T., Ashbolt N.J. (2013). Eukaryotic diversity in premise drinking water using 18S rDNA sequencing: Implications for health risks. Environ. Sci. Pollut. Res. Int..

[46] Schuster F.L., Visvesvara G.S. (2004). Free-living amoebae as opportunistic and non-opportunistic pathogens of humans and animals. Int. J. Parasitol..

[47] Meyer C., Desalme D., Bernard N., Binet P., Toussaint M.-L., Gilbert D. (2013). Using testate amoeba as potential biointegrators of atmospheric deposition of phenanthrene (polycyclic aromatic hydrocarbon) on “moss/soil interface-testate amoeba community” microecosystems. Ecotoxicology.

[48] Aguilar M., Richardson E., Tan B., Walker G., Dunfield P., Bass D., Nesbø C., Foght J., Dacks J.B. (2016). Next-generation Sequencing Assessment of Eukaryotic Diversity in Oil Sands Tailings Ponds Sediments and Surface Water. J. Eukaryot. Microbiol..

[49] Walker G., Dacks J.B., Martin Embley T. (2006). Ultrastructural Description of Breviata anathema, N. Gen., N. Sp., the Organism Previously Studied as “Mastigamoeba invertens”. J. Eukaryot. Microbiol..

[50] Bass D., Czech L., Williams B., Berney C., Dunthorn M., Mahé F., Torruella G., Stentiford G.D., Williams T.A. (2018). Clarifying the Relationships between Microsporidia and Cryptomycota. J. Eukaryot. Microbiol..

[51] Howe A.T., Bass D., Vickerman K., Chao E.E., Cavalier-Smith T. (2009). Phylogeny, Taxonomy, and Astounding Genetic Diversity of Glissomonadida ord. nov., The Dominant Gliding Zooflagellates in Soil (Protozoa: Cercozoa). Protist.

[52] Rogerson A., Berger J. (1982). Ultrastructural Modification of the Ciliate Protozoan, Colpidium colpoda, Following Chronic Exposure to Partially Degraded Crude Oil on JSTOR. BioScience.

[53] Kota S., Borden R.C., Barlaz M.A. (1999). Influence of protozoan grazing on contaminant biodegradation. FEMS Microbiol. Ecol..

[54] Tso S.-F., Taghon G.L. (2006). Protozoan grazing increases mineralization of naphthalene in marine sediment. Microb. Ecol..

[55] Gilbert D., Jakobsen H.H., Winding A., Mayer P. (2014). Co-transport of polycyclic aromatic hydrocarbons by motile microorganisms leads to enhanced mass transfer under diffusive conditions. Environ. Sci. Technol..

[56] Marentette J.R., Sarty K., Cowie A.M., Frank R.A., Hewitt L.M., Parrott J.L., Martyniuk C.J. (2017). Molecular responses of Walleye (Sander vitreus) embryos to naphthenic acid fraction components extracted from fresh oil sands process-affected water. Aquat. Toxicol..

[57] Alharbi H.A., Alcorn J., Al-Mousa A., Giesy J.P., Wiseman S.B. (2017). Toxicokinetics and toxicodynamics of chlorpyrifos is altered in embryos of Japanese medaka exposed to oil sands process-affected water: Evidence for inhibition of P-glycoprotein. J. Appl. Toxicol..

[58] Peng H., Sun J., Alharbi H.A., Jones P.D., Giesy J.P., Wiseman S.B. (2016). Peroxisome Proliferator-Activated Receptor γ is a Sensitive Target for Oil Sands Process-affected Water: Effects on Adipogenesis and Identification of Ligands. Environ. Sci. Technol..

[59] Leung S.S., MacKinnon M.D., Smith R.E.H. (2003). The ecological effects of naphthenic acids and salts on phytoplankton from the Athabasca oil sands region. Aquat. Toxicol..

[60] Woodward F.I. (2007). Global primary production. Curr. Biol..

[61] Ruffell S.E., Frank R.A., Woodworth A.P., Bragg L.M., Bauer A.E., Deeth L.E., Müller K.M., Farwell A.J., Dixon D.G., Servos M.R. (2016). Assessing the bioremediation potential of algal species indigenous to oil sands process-affected waters on mixtures of oil sands acid extractable organics. Ecotoxicol. Environ. Saf..

[62] Graham L.E., Graham J.M., Cook M.E., Wilcox L.W. (2009). Algae.

[63] Dompierre K.A., Barbour S.L. (2016). Characterization of physical mass transport through oil sands fluid fine tailings in an end pit lake: A multi-tracer study. J. Contam. Hydrol..

[64] White K.B., Liber K. (2018). Early chemical and toxicological risk characterization of inorganic constituents in surface water from the Canadian oil sands first large-scale end pit lake. Chemosphere.

[65] Tedford E., Halferdahl G., Pieters R., Lawrence G.A. (2018). Temporal variations in turbidity in an oil sands pit lake. Environ. Fluid Mech..

[66] Risacher F.F., Morris P.K., Arriaga D., Goad C., Nelson T.C., Slater G.F., Warren L.A. (2018). The interplay of methane and ammonia as key oxygen consuming constituents in early stage development of Base Mine Lake, the first demonstration oil sands pit lake. Appl. Geochem..

[67] Rekik A., Denis M., Dugenne M., Maalej S., Ayadi H. (2015). Journal of Oceanography, Research and Data.

[68] Stoeck T., Edgcomb V. (2010). Role of Protists in Microbial Interactions with Hydrocarbons. Handbook of Hydrocarbon and Lipid Microbiology.

[69] Caron D.A. (2016). Mixotrophy stirs up our understanding of marine food webs. Proc. Natl. Acad. Sci. USA.

[70] Foissner W. (2016). Protists as bioindicators in activated sludge: Identification, ecology and future needs. Eur. J. Protistol..

[71] Howe A.T., Bass D., Chao E.E., Cavalier-Smith T. (2011). New Genera, Species, and Improved Phylogeny of Glissomonadida (Cercozoa). Protist.

[72] Alberta Agriculture and Forestry (2017). A Review of the 2016 Horse River Wildfire Alberta Agriculture and Forestry Preparedness and Response.

[73] Zhang Q., Carroll J.J., Dixon A.J., Anastasio C. (2002). Aircraft Measurements of Nitrogen and Phosphorus in and around the Lake Tahoe Basin: Implications for Possible Sources of Atmospheric Pollutants to Lake Tahoe. Environ. Sci. Technol..

[74] Rust A.J., Hogue T.S., Saxe S., McCray J. (2018). Post-fire water-quality response in the western United States. Int. J. Wildl. Fire.

[75] Vicars W.C., Sickman J.O., Ziemann P.J. (2010). Atmospheric phosphorus deposition at a montane site: Size distribution, effects of wildfire, and ecological implications. Atmos. Environ..

